# A module of inflammatory cytokines defines resistance of colorectal cancer to EGFR inhibitors

**DOI:** 10.18632/oncotarget.12354

**Published:** 2016-09-30

**Authors:** Valerio Gelfo, Maria Teresa Rodia, Michela Pucci, Massimiliano Dall'Ora, Spartaco Santi, Rossella Solmi, Lee Roth, Moshit Lindzen, Massimiliano Bonafè, Andrea Bertotti, Elisabetta Caramelli, Pier-Luigi Lollini, Livio Trusolino, Yosef Yarden, Gabriele D'Uva, Mattia Lauriola

**Affiliations:** ^1^ Department of Experimental, Diagnostic and Specialty Medicine (DIMES), University of Bologna, Bologna, Italy; ^2^ Center for Applied Biomedical Research (CRBA) S. Orsola-Malpighi University Hospital, University of Bologna, Bologna, Italy; ^3^ Institute of Molecular Genetics, National Research Council of Italy, Bologna, Italy; ^4^ Laboratory of Musculoskeletal Cell Biology, IOR-IRCCS, Bologna, Italy; ^5^ Department of Biological Regulation, Weizmann Institute of Science, Rehovot, Israel; ^6^ Candiolo Cancer Institute-Fondazione del Piemonte per l'Oncologia (FPO), IRCCS, Department of Oncology, University of Torino, Candiolo, Italy; ^7^ Scientific and Technology Pole, IRCCS MultiMedica, Milan, Italy

**Keywords:** EGFR, transcriptional response, colon cancer, resistance, cetuximab

## Abstract

Epidermal Growth Factor Receptor (EGFR) activates a robust signalling network to which colon cancer tumours often become addicted. Cetuximab, one of the monoclonal antibodies targeting this pathway, is employed to treat patients with colorectal cancer. However, many patients are intrinsically refractory to this treatment, and those who respond develop secondary resistance along time. Mechanisms of cancer cell resistance include either acquisition of new mutations or non genomic activation of alternative signalling routes. In this study, we employed a colon cancer model to assess potential mechanisms driving resistance to cetuximab. Resistant cells displayed increased ability to grow in suspension as colonspheres and this phenotype was associated with poorly organized structures. Factors secreted from resistant cells were causally involved in sustaining resistance, indeed administration to parental cells of conditioned medium collected from resistant cells was sufficient to reduce cetuximab efficacy. Among secreted factors, we report herein that a signature of inflammatory cytokines, including *IL1A*, *IL1B* and *IL8*, which are produced following EGFR pathway activation, was associated with the acquisition of an unresponsive phenotype to cetuximab *in vitro*. This signature correlated with lack of response to EGFR targeting also in patient-derived tumour xenografts. Collectively, these results highlight the contribution of inflammatory cytokines to reduced sensitivity to EGFR blockade and suggest that inhibition of this panel of cytokines in combination with cetuximab might yield an effective treatment strategy for CRC patients refractory to anti-EGFR targeting.

## INTRODUCTION

Epidermal Growth Factor Receptor (EGFR) was the first member of the ERBB family to be discovered, and later found to be directly mutated or overexpressed in solid tumours. The roles played by EGFR proved to be central in colorectal cancer patients, who often present an addiction to this pathway [1]. EGFR signalling appears to be propagated downstream through multiple effectors, with a main vertical transmission of the signal. Nevertheless, in the last decade, the development of high throughput technologies described the ERBB family as a systemic network that follows the principles of network theories [2, 3], characterized by multiple levels of regulatory loops, namely feed-back and feed-forward loops [4]. The integration and often intertwining negative- and positive-feedback circuits help maintaining appropriate quantitative and dynamic relationships between inputs (growth factor stimuli) and outputs (cellular phenotype), allowing a fast and stable attainment of a new steady state [5]. This ensures that most cellular parameters stay under tight control within a narrow range and around a certain optimal level. Feedback deregulation is thus often responsible for diseases, hence characterization of either positive or negative feedback, including transcription-mediated processes, has been extensively investigated in the last decade [6, 7].

The development of neutralizing antibodies targeting EGFR, such as cetuximab and panitumumab, created a breakthrough in metastatic colorectal cancer (mCRC) treatment [8, 9]. EGFR antibodies in combination with chemotherapy prolong survival in subjects with mCRC and are a standard component of therapy of such individuals [9]. The genetic evaluation for *KRAS* and *NRAS* mutations currently represents the main clinical criterion predicting treatment efficacy, since mutations in these genes foresees an individual's intrinsic resistance to the monoclonal antibodies [10]. However, unfortunately, many subjects with *KRAS*/*NRAS* wild-type mCRC display de novo resistance, and those who initially respond ultimately acquire secondary resistance to these agents [11, 12]. All these clinical observations require a deeper understanding of the mechanisms involved in the failure to intercept EGFR, which leads to drug resistance. Thus, mechanistic studies in this direction will endorse the development of more effective therapeutic approaches.

Resistance to EGFR blockade may have a genetic basis, including oncogenic activation of downstream or parallel signalling pathways that substitute for EGFR inhibition [13], but may also rely on plastic, reversible traits induced by drug pressure [14], such as compensatory activation of biochemical feedback circuits and transcriptional modifications [15–17]. In this study we employed CRC cell line to explore the plastic phenotype of cellular adaptation to prolonged cetuximab treatment. We found that resistance to EGFR targeting drugs results in the up-regulation of a signature of inflammatory cytokines, namely *IL1A*, *IL1B* and *IL8*. The association between reduced sensitivity to anti-EGFR antibodies and increased expression of this inflammatory signature was confirmed in patients’ data and specimens.

## RESULTS

### Long-term exposure to antibody- or small molecule-mediated EGFR inhibition leads to the emergence of cross-resistant cell lines

**Figure 1:**
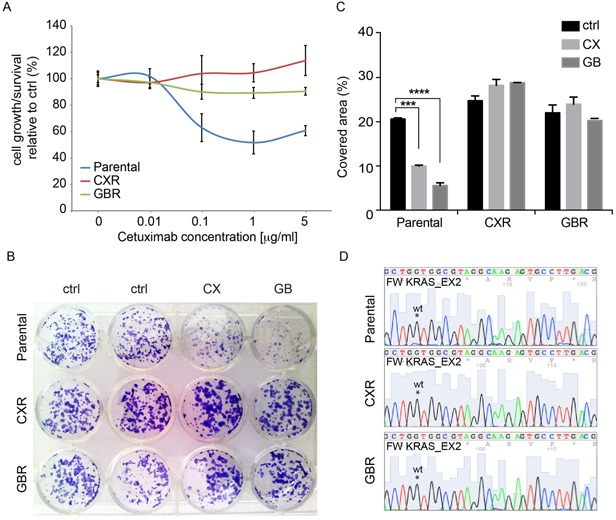
Establishment of colorectal cells resistant to cetuximab Human colorectal cancer Caco-2 cells were made resistant to cetuximab (CX) and gefitinib (GB) by continuous exposure to increasing doses of drug and maintained with 2 μg/ml of CX and 1 μM of GB. **A.** Cell proliferation analysis by Alamar assay of parental, cetuximab-resistant (CXR) and gefitinib-resistant (GBR) cells following treatments with increasing concentration of cetuximab in medium containing 1% serum for 72h. The graphic represents the relative proliferation/viability of the cells following 72 hours of treatment related to control; **B-C.** Colony formation assay of parental, cetuximab-resistant (CXR) and gefitinib-resistant (GBR) cells. Cells were grown in absence or presence of CX (1μg/ml) and GB (1 μM) for 10 days in medium containing 1% of serum, then fixed, stained with Crystal Violet and photographed. Representative figures and quantification of the covered areas by ImageJ is provided in B and C, respectively. The statistic was calculated by 2-way ANOVA, *** P<0.0001, ** P<0.01. These experiments were repeated at least three times. **D.** Sequence analyses of KRAS Exon 2, showing the wild type sequence detected in parental, CXR and GBR cells.

We established cetuximab-resistant cells from the human colorectal cell line Caco-2, which is wild-type for *KRAS* and *BRAF*, and dependent on EGFR as a mitogenic stimulus, as previously characterized in our laboratory [18]. Caco-2 cells were made resistant to cetuximab (CX) or the EGFR small-molecule inhibitor gefinitib (GB), by continuous exposure over six months to increasing concentrations of the drugs, ranging from 10 ng/ml to 20 μg/ml in the case of CX and from 1nM to 10μM for GB. Notably, the cells were kept under CX (2 μg/ml) or GB treatment (1μM), while propagating. We first confirmed resistance, by measuring cell growth using the Alamar assay. Caco-2 parental cells and CX or GB resistant cells, hereinafter referred as parental, CXR and GBR, respectively, were treated with increasing concentrations of CX for 72-96 h and their proliferation was compared to cells treated with vehicle only, DMSO or PBS (Figure 1A). While parental cells displayed growth inhibition under CX treatment, with maximal inhibition achieved at 1 to 5 μg/ml of the antibody, the resistant sub-line CXR displayed undisturbed viability under CX treatment, confirming acquired resistance. Similarly, the GBR cells, which exhibited resistance to GB treatment, remained fully viable under CX treatment, suggesting a cross-resistance of GB-resistant cells to CX (Figure 1A).

We next performed a long-term clonogenic assay, by seeding a very low number of cells in twelve well plates with the indicated treatments. After 10 days, the cells were fixed and stained with crystal violet, and clonogenic cell growth was evaluated by measuring the portion of covered area of the plate. In this assay, parental cells displayed a ~50% and ~70% growth inhibition upon CX and GB treatment, respectively (Figure 1B, C). In contrast, no change in clonogenic cell growth was detected in CXR and GBR cells, supporting the previous observation of an acquired cross-resistance. Finally, we performed the sequence analyses of KRAS exon 2, codon 12, in order to test whether acquisition of KRAS mutation could explain the observed resistant phenotype. Interestingly, none of the CXR or GBR cells displayed acquired mutation (Figure 1D).

Overall, our Caco-2 *in vitro* model suggests a mechanism of resistance to EGFR targeted therapies shared by monoclonal antibodies and small tyrosine kinase inhibitors, independent from acquisition of KRAS mutation.

### Resistant cells display anchorage-independent growth as spheroids

**Figure 2:**
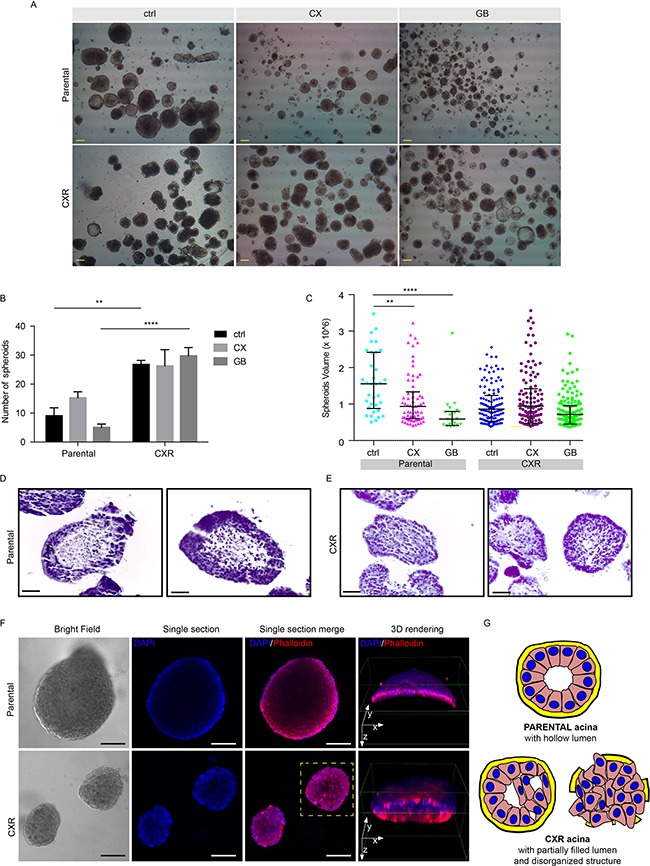
Cetuximab resistant cells displayed increased ability to growth in suspension as colonspheres We investigated the morphology of Caco-2 parental, CXR and GBR cells when forced to grow in suspension. **A.** 4X magnification of Caco-2 producing “spheroid-like” structures, under the indicated treatments. Scale bar 100μm; **B.** Number of filled spheroids presented as average ± S.E.M. 2-way ANOVA with Bonferroni Test, ** p<0.01; ****p<0.0001; **C.** Quantification of spheroids sizes measurements under the indicated treatments in 5% FBS (EGF 10ng/ml, CX 1μg/ml and GB 1μM) is presented as dots plot. Each dot represents the quantification of a single spheroid (n= 117 for Parental cells, n=332 for CXR). Bar represents volume averages ± S.E.M, 1-way ANOVA, ** p<0.01; ****p<0.0001; **D-E.** Analysis of Caco-2 parental and CXR paraffin-embedded spheroid morphology by H&E staining. Scale bar 50 μm; **F.** Single section passing through the maximum diameter of spheroids and 3D confocal morphology of parental and CXR spheroids. Left panel: bright field imaging; central panels: DAPI and Phalloidin confocal microscopy; right panel: three-dimensional reconstructions of both signals, obtained by rotating the y axes and cutting the lower part of the spheroids (green lines) to observe the inner structures; scale bar 100 μm; **G.** Illustration depicting a schematic representation of the parental and CXR spheroid morphology.

The ability to grow in suspension is a hallmark of the neoplastic phenotype. Notably, only a small percentage, about 0.5% of Caco-2 parental cells displayed the ability to grow in suspension and form spheroid-like structures. In contrast, more then 1.2% of CXR cells displayed the ability to form spheroids (Figure 2A, B). Statistically, parental cell spheroids were fewer and of larger size, when compared to CXR cells (Figure 2B, C). CX and GB treatments decreased sphere volumes in parental cells (Figure 2C), while in CXR cells did not induce effects in terms of either spheroid size or number, supporting the lack of sensitivity of these cells to EGFR-targeting drugs (Figure 2A–C). Next, we investigated the morphology of the spheroids both by embedding them into paraffin blocks, preparing 8-10 μm slides and staining with hematoxylin-eosin (H&E) (Figure 2D) or performing confocal microscopy analysis (Figure 2F). Parental cells displayed a well-organized architecture, with multiple layers of nuclei polarized on the external region and a hollow lumen filled with cell debris and matrix, as shown in Figure 2D, 2F and illustrated by Figure 2G. On the other hand, CXR cells displayed poorly organized structures, with sparse nuclei in the entire volume and smaller and filled lumen (Figure 2E, F). Furthermore, 3D rendering elaboration of the actin and nuclei signals, obtained by rotating the y axes and cutting the lower part of the spheroids helped to visualize the spheroid lumen, which appeared more filled in the resistant cells. Summarizing, the CXR cells acquired a robust ability to grow in suspension. Parental spheroids are well organized and display hollow lumens, whereas CXR spheroids are smaller, poorly organized and filled or partially filled with cells. These findings support the notion that the adaptation to cetuximab leads to resistant cells characterized by a more malignant phenotype, which enables the cells to grow in suspension.

### A module of inflammatory cytokines is induced in cetuximab resistant cells

**Figure 3:**
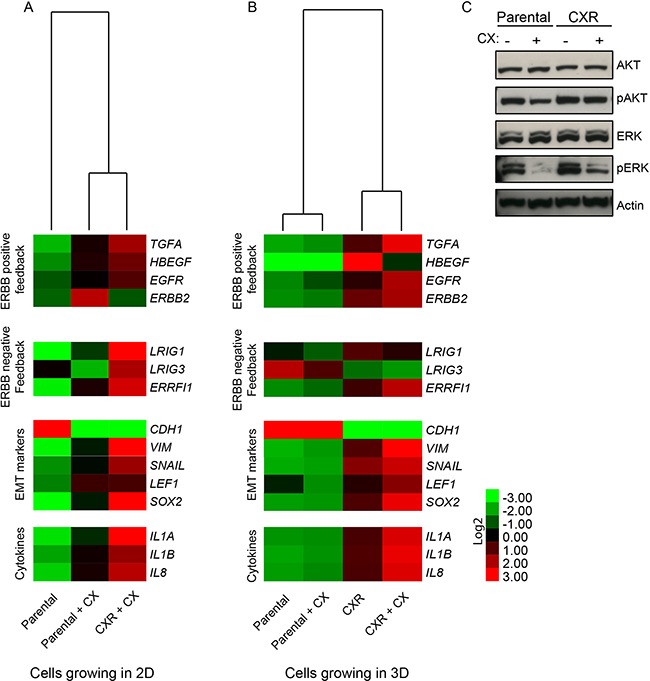
Gene expression analysis of parental and CXR cells **A-B.** PCR analysis of parental or cetuximab resistant (CXR) cells growing as monolayer (A) or as spheroids (B) were collected, both under CX (2μg/ml) treatment or regular medium conditions. A set of genes probing EGFR positive/negative feedback loop, EMT phenotype and inflammatory cytokines were analysed and displayed as heatmap. **C.** Western blot analysis of phospho-AKT (pAKT), AKT, phospho-ERK (pERK) and ERK levels in Caco-2 parental and CXR cells treated over-night with and without cetuximab. Actin served as a loading control.

**Figure 4:**
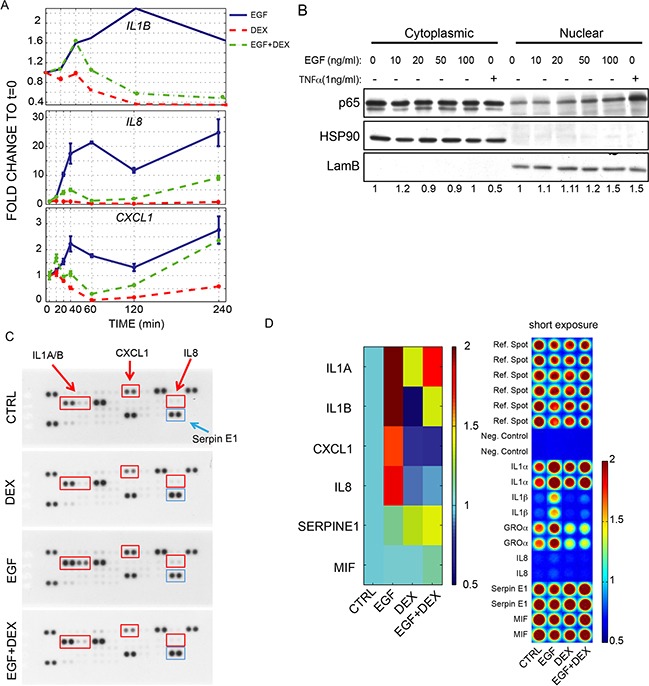
EGFR activation controls the production of a module of inflammatory cytokines in MCF10A **A.** Expression levels of the module of inflammatory cytokines (*IL1B* and *IL8* and *CXCL1*) analysed by Real-Time PCR in MCF10A following EGF (10ng/ml) or DEX (100nM) administration, alone or in combination, over a time course of 4h; **B.** Western blot of NF-kB in cytoplasmic and nuclear fraction of MCF10A cells following administration of increasing doses of EGF (from 1 to 100ng/ml). TNFα (1ng/ml) treatment was used as positive control for NF-kB (p65) activation and nuclear translocation. Laminin B and HSP90 were used as loading control for the nuclear and cytosolic fraction, respectively; **C-D.** Cytokine array of MCF10A cells following administration of EGF (10ng/ml), DEX (100nM), alone or in combination. Representative pictures are provided in C. Quantification of chemioluminescence along with images of normalized spots are provided in D.

Secreted growth factors and cytokines have been shown to contribute to drug resistance by imparting compensatory survival cues [16,19,20]. We sought to analyse gene expression of parental and resistant Caco-2 cells, in the presence or absence of cetuximab. The analysis included critical components of positive ERBB feedback regulatory loops (such as the EGFR ligands *TGFA*, *HBEGF*), which were recently found to be involved in cetuximab resistance [21,22], and of negative ERBB feedback loops, namely inhibitors of EGFR/ERBB signaling, such as *LRIG1*, *LRIG3* and *ERRFI1* [23,24]. To provide further functional annotation, we also interrogated markers of the Epithelial to Mesenchymal Transition (EMT), such as E-cadherin, vimentin, *SNAIL*, *LEF1* and *SOX2* (a transcription factor associated with stemness) [25]. Finally, we analysed the levels of inflammatory cytokines (*IL1A*, *IL1B*, *IL8*), which were previously demonstrated to play a role in carcinogenesis [26], but whose involvement in resistance to cetuximab was not reported. The results are displayed in Figure 3A as heat-maps, with red boxes corresponding to relatively high expression of the respective transcript and green boxes corresponding to low expression. Under monolayer conditions, the autocrine ligands *HBEGF* and *TGFA* were slightly increased upon cetuximab administration and up-regulated in cetuximab-resistant cells, which also displayed increased levels of negative feedback regulators (Figure 3A). In addition, resistant cells also featured increased levels of markers of epithelial-mesenchymal transition and stem-like features, with reduced expression of *CDH1* (E-cadherin) and increased expression of vimentin, the EMT inducer *SNAIL*, and the stem-cell transcription factors *LEF1* and *SOX2* (Figure 3A). Finally, EGFR inhibition by cetuximab in parental cells led to a slight increase in expression of *IL1A*, *IL1B* and *IL8*; and these cytokines were markedly overexpressed in cetuximab-resistant cells (Figure 3A). Most of the transcriptional modulations that occurred in 2D resistant cells could be also observed in resistant spheroids (Figure 3B), indicating maintenance of these traits irrespective of culture conditions. Acquired production of the positive feedback components, such as *HBEGF* and simultaneous inhibition of EGFR by negative feedback might reflect a shift toward HER2 heterodimer activation, as previously reported [15,27]. Of note, spheroids from parental cells did not experience any obvious transcriptional reprogramming following exposure to cetuximab, apart from increased expression of some EGFR negative regulators. The weak transcriptional consequences of EGFR blockade in parental spheroids could be related to initial selection of cells growing in suspension, which might have enriched for cells that are more resistant to pro-apoptotic insults, including anchorage-independent growth and EGFR blockade. Analyses of the signalling pathways downstream to EGFR helped us to characterize signalling differences between parental and CXR cells. Indeed, while parental cells responded to CX treatment, by decreasing both phosphorylation of AKT and ERK (Figure 3C), resistant cells appeared not sensitive to CX inhibition on AKT phosphorylation. Interestingly the basal level of pERK was in general higher in resistant cells, and CX still displayed inhibitory capability on pERK, although less effective (Figure 3C). Collectively, this analysis shows that the resistant phenotype is accompanied by increased expression of inflammatory cytokines and EGF-like growth factors, feedback activation of EGFR negative regulators, and EMT/stem-like features. These data are further supported by the evidence that EGFR activation in epithelial cells, such as MCF10A human mammary cells, immediately produces a module of inflammatory cytokines, namely *IL1B, IL8* and *CXCL1*, by active transcriptional production (Figure 4A). MCF10A cells are highly dependent on EGFR pathway, thus it represents a useful tool to study EGFR signalling, avoiding perturbations derived from tumor transformation. Furthermore, co-treatment with Dexamethasone (DEX), a global inhibitor of the EGFR transcriptional response [7,28], dampened this production, pointing to a direct involvement of the EGFR pathway in the transcriptional induction of inflammatory mediators (Figure 4A). Because EGF simultaneously up-regulated several inflammatory cytokines and because this effect was abrogated by DEX, which is a powerful anti-inflammatory agent mainly acting through repression of NF-κB [29], we assumed that the inflammation-regulating transcription factor NF-κB is activated by EGF in mammary epithelial cells. In line with this model, we observed a dose-dependent activation of NF-κB (p65) by EGF (Figure 4B). To extend these observations to the protein level and also to assay additional cytokines, we utilized a cytokine array (Figure 4C, D). This analysis detected high basal levels of IL1A in untreated cells, which was increased following EGF stimulation and dampened by the DEX co-treatment. Interestingly, IL1B, IL8 and CXCL1 displayed very low basal levels with strong induction upon the growth factor stimulus, and once again the anti-inflammatory action of DEX was able to block production of these cytokines, in line with our previous observations [7].

### Secreted factors from cetuximab resistant cells are causally involved in sustaining resistance to cetuximab

**Figure 5:**
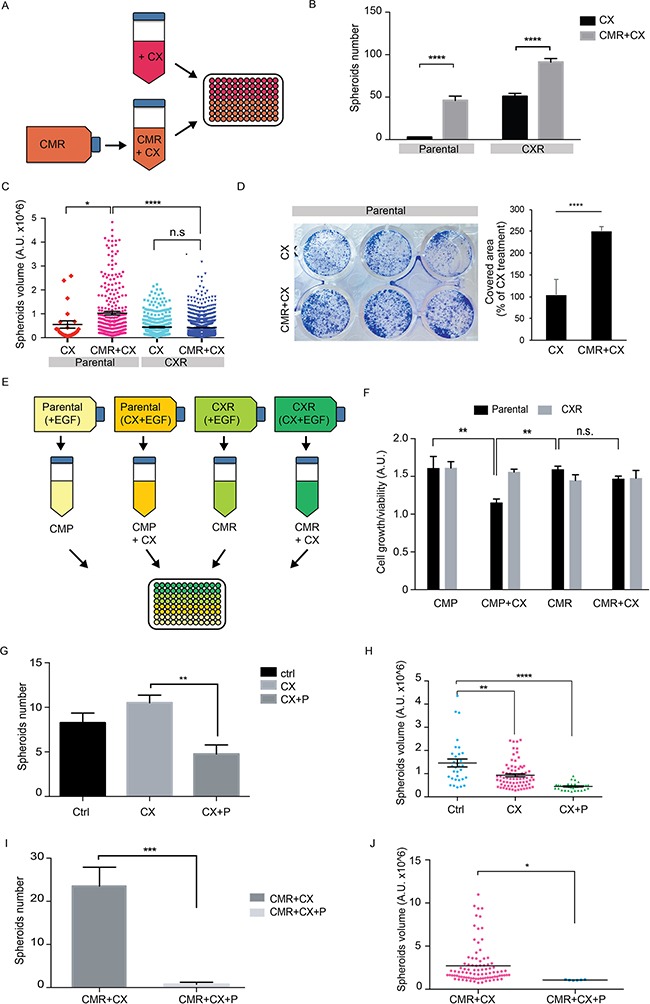
Secreted factors from CX resistant cells reduces cetuximab sensitivity on parental cells **A.** Schematic diagram showing the modality of obtaining conditioned medium from CXR cells (CMR), which was subsequently used for treatments. CMR was collected from CXR Caco2 cells cultured for 72h in presence of cetuximab (2 μg/ml); **B-C.** Spheroid assay of parental and CXR cells cultured in fresh medium or CMR, both supplemented with EGF (10 ng/ml) and cetuximab (2 μg/ml). Pictures at 4x magnification of at least four not overlapping fields were taken after 10 days and spheroids number and volume were measured. Quantification of spheroids number (mean ± s.e.m) is provided in B. Quantification of volume of each spheroid and the mean value for each treatment is provided in C. * p< 0.05 **** p< 0.0001, n.s.: not significant (two-way ANOVA); **D.** Colony formation assay of parental cells seeded with fresh medium or conditioned medium from CXR cells (CMR), both supplemented with Cetuximab (2 μg/ml). Representative images of crystal violet staining are provided in the left panel. Quantification of the relative covered area, displayed as percentage of conditioned medium treatment (mean ± s.e.m), is provided in the right panel. p<0.00026 (t-test). **E.** Scheme depicting the second modality of conditioned media production from Parental or CXR Caco-2 cells. In these settings the conditioned media was collected from parental and CXR cells cultured for 72 hours in full medium supplemented with EGF (10 ng/ml) alone or in combination with cetuximab (2 μg/ml); **F.** Parental and CXR cells were treated with the conditioned medium obtained as described in E. Cell growth/viability was measured with Alamar blue assay after 96 hours (in the graph mean ± s.e.m, arbitrary unit). ** p<0.01, n.s.: not significant (two-way ANOVA). **G-H.** Spheroid assay of parental cells cultured in fresh medium, supplemented with EGF (10 ng/ml) and cetuximab (2 μg/ml) plus parthenolide (1μM). Pictures at 4x magnification of at least four not overlapping fields were taken after 10 days and spheroids number and volume were measured. Quantification of spheroids number (mean ± s.e.m) is provided in G. Quantification of spheroid volumes along with the mean value for each treatment is provided in H. ** p< 0.01 **** p< 0.0001 (one -way ANOVA); **I-J.** Spheroid assay of parental cells cultured in fresh medium, or conditioned medium from CXR cells (CMR) supplemented with EGF (10 ng/ml) and cetuximab (2 μg/ml), alone or in combination with parthenolide (10 μM). Pictures at 4x magnification of at least four not overlapping fields were taken after 10 days and spheroids number and volume were measured. Quantification of spheroids number (mean ± s.e.m) and volumes is provided in I and J, respectively. * p< 0.05 ** p< 0.01 **** p< 0.0001 (one -way ANOVA).

To analyse if changes in factors secreted from resistant cells are causally involved in sustaining resistance, we first tested the cetuximab efficacy on spheroidogenesis and colony formation ability following administration of conditioned media (CM) from cetuximab resistant cells (CMR) (Figure 5A). The CM from CXR cells, hereinafter referred as CMR, displayed effect both on spheroids number and size, promoting a six-fold increase in number (Figure 5B) and two-fold increase in sphere dimension (Figure 5C). Administration of CMR to resistant cells also increased spheroidogenesis in terms of number (Figure 5B), but not in terms of volume (Figure 5C). We further tested the response to cetuximab in the presence of conditioned medium from resistant cells in a colony forming assay. Again, by measuring the clonogenic proliferation by crystal violet staining, the inhibitory ability of cetuximab was impaired by 60% when parental cells were kept in the presence of CMR (Figure 5D). Finally, we collected the conditioned medium both from parental (CMP) and CXR cells (CMR), as depicted in Figure 5F. The proliferation experiments in these conditions show that the efficacy of CX is maintained when combined with conditioned medium collected from parental cells. In contrast, simultaneous administration of conditioned medium from CXR cells reduced the inhibitory activity of cetuximab on cell proliferation (Figure 5F).

All these data pointed to the presence of secreted molecules from CXR cells to the CM, which decreased cetuximab efficacy and acted as strong mitogenic stimuli. Interestingly, the conditioned medium of parental cells did not affect cetuximab response in sensitive cells; suggesting that soluble molecules specifically secreted by CXR cells are sufficient to overcome the response to cetuximab in otherwise sensitive cells.

Next we tested whether the clonogenic property induced by CMR was mediated by the activation of NF-κB. Indeed, NF-κB is a well-known hub mediator for inflammation and tumor growth. We employed parthenolide, a natural NF-κB inhibitor, previously reported to inhibit mammospheres formation induced by TNF-alpha [30]. Parthenolide treatment decreased the colonspheres forming capacity of parental cells growing under regular medium (Figure 5G, H). Importantly, parthenolide was able to prevent the spheroidogenesis induced by CMR conditioned medium (Figure 5I, J). These results support the hypothesis that NF-kB is a key player in resistance to CX treatment, likely for its ability of inducing pro-inflammatory cytokines production (such as IL1A, IL1B and IL8), which in turn activates a NF-κB dependent feed-forward loop, as reported in pancreatic tumors [31].

### A module of inflammatory cytokines predicts resistance to cetuximab in colorectal patients

**Figure 6:**
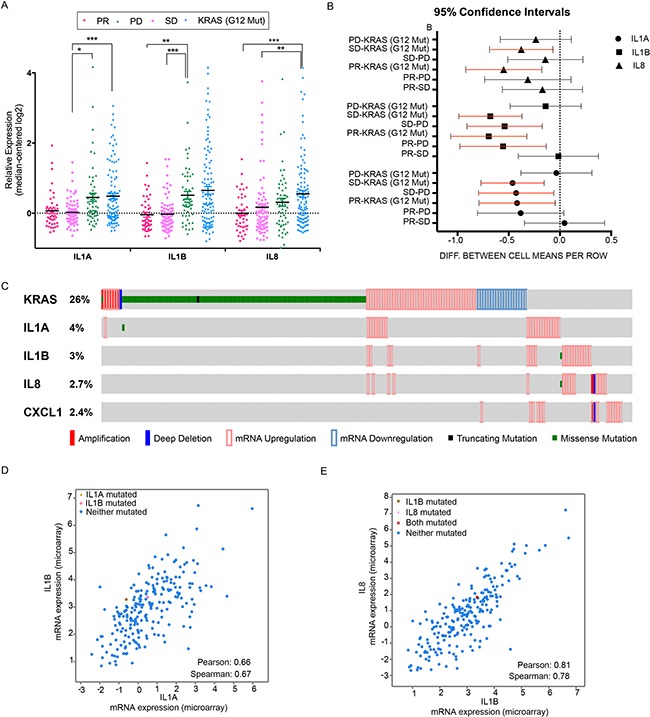
A panel of inflammatory cytokines correlates with cetuximab response in colorectal patients **A.** Expression analysis of *IL1A, IL1B* and *IL8* in colorectal quadruple wild type (wild type for *KRAS*, *BRAF*, *NRAS* and *PIK3CA*) tumors, subdivided by response to Cetuximab therapy: disease regression (PR), disease stabilization (SD), disease progression (PD). The KRAS (G12 Mut) group was included as control for lack of response to CX; **B.** Forest plot summarizing the differences between the indicated groups in the data set of colon cancer patients. Each line represents the 95% confidence interval corresponding to the listed comparisons. Note that the red lines indicate statistical significance; **C.** RNAseq data of colon cancer samples from TCGA Colorectal Adenocarcinoma (631 samples from 627 patients). **D-E.** Co-expression analysis from TGCA datasets, of *IL1A* and *IL1B* (D) and of *IL8* and *IL1B* (E). Statistically significant values were computed and Pearson and Spearman correlation values are provided.

Our data *in vitro* pointed out the increased expression of a panel of inflammatory cytokines in cells resistant to cetuximab. We therefore decided to analyse the panel of selected cytokines in patients colorectal tumorgraft. Gene expression information was analysed in colorectal tumorgrafts from 98 patients with wild type *KRAS*, *BRAF*, *NRAS*, and *PIK3CA* genotypes (“quadruple negative” tumors) and from 61 individuals with KRAS (G12) mutation. *KRAS* G12 mutation leads to a constitutively activated K-Ras protein, which confers an intrinsic resistance to EGFR blockade. WT quadruple negative tumorgrafts were tested for cetuximab response, as described by Bertotti [32]. In this condition, the human stroma is supposed to be substituted by murine components; therefore, this analysis covers only receptors and autocrine ligands expressed by cancer cells. Interestingly, we observed an inverse association between elevation of inflammatory cytokines *IL1A*, *IL1B* and *IL8* and the overall response to cetuximab (Figure 6A). In accordance with our *in vitro* data, *IL1A*, *IL1B* and *IL8* were overexpressed in tumorgrafts that proved to be resistant to EGFR blockade (tumor volume increase of at least 35% compared to the initial, pre-treatment volume). Furthermore, intermediate *IL8* expression levels were observed in a group with limited sensitivity to cetuximab, with tumour volume changes between 35% increase and 50% reduction, which is considered as stable disease (SD) (Figure 6A, B). Interestingly, the pattern of increased expression of this module of inflammatory cytokines was maintained in the group of *KRAS* mutant tumours, which by definition are resistant to treatment. These results suggest that gradual tumour adaptation to EGFR blockade might be associated with up-regulation of the module of inflammatory cytokines, which might be responsible for tumor plasticity and activation of compensatory pathway, thus overcoming EGFR inhibition.

We next tested the expression of the module of the identified cytokines *IL1A*, *IL1B* and *IL8* on data generated by the TCGA Research Network (http://cancergenome.nih.gov/), which encompasses 631 patients’ tumour samples. Specifically, we interrogated the Colorectal Adenocarcinoma subset (TGCA Provisional) and filtered mRNA expression levels with reported expression z-Scores (RNA Vs RSEM) >= 1.5, as normalized against the expression distribution of all gene tumours that are diploid for the corresponding gene. By doing so, we identified about 11% of tumors (67 out of 631 samples) displaying an alteration on the probed cytokines panel (Figure 6C). We also interrogated the co-expression of the reported genes on the same data set (17157 genes from 244 colorectal adenocarcinoma cases); strikingly, *IL1B*, *IL8* and *CXCL1* display the highest correlation with *IL1A*, with a Pearson correlation of 0.66, 0.55 and 0.51 respectively. We also observed a correlation between *IL1B* and *IL8* (Figure 6E), which reaches the highly significant value of 0.78 Pearson and 0.81 Spearman. These data suggest a common regulation of the reported cytokines, which might be driven by shared regulators, such as the inflammatory Transcription Factor NF-κB. To sum up, these results propose that about 11 % of the colon cancer population might benefit by intercepting NF-κB along with the detected cytokines. Notably, TGCA data show a lack of correlations of the reported cytokines with KRAS mutation and our in vitro model confirms a RAS wild-type status in the resistant condition.

## DISCUSSION

Our study points to the production of soluble cytokines IL1A, IL1B and IL8, as markers of a poor response to cetuximab and as potential key factors in CRC tumor adaptation to cetuximab pressure. These data suggest to evaluate this panel of inflammatory cytokines in patients’ tumour specimens before initiating EGFR-treatment, since it could represent an additional criteria predicting therapy efficacy.

**Figure 7:**
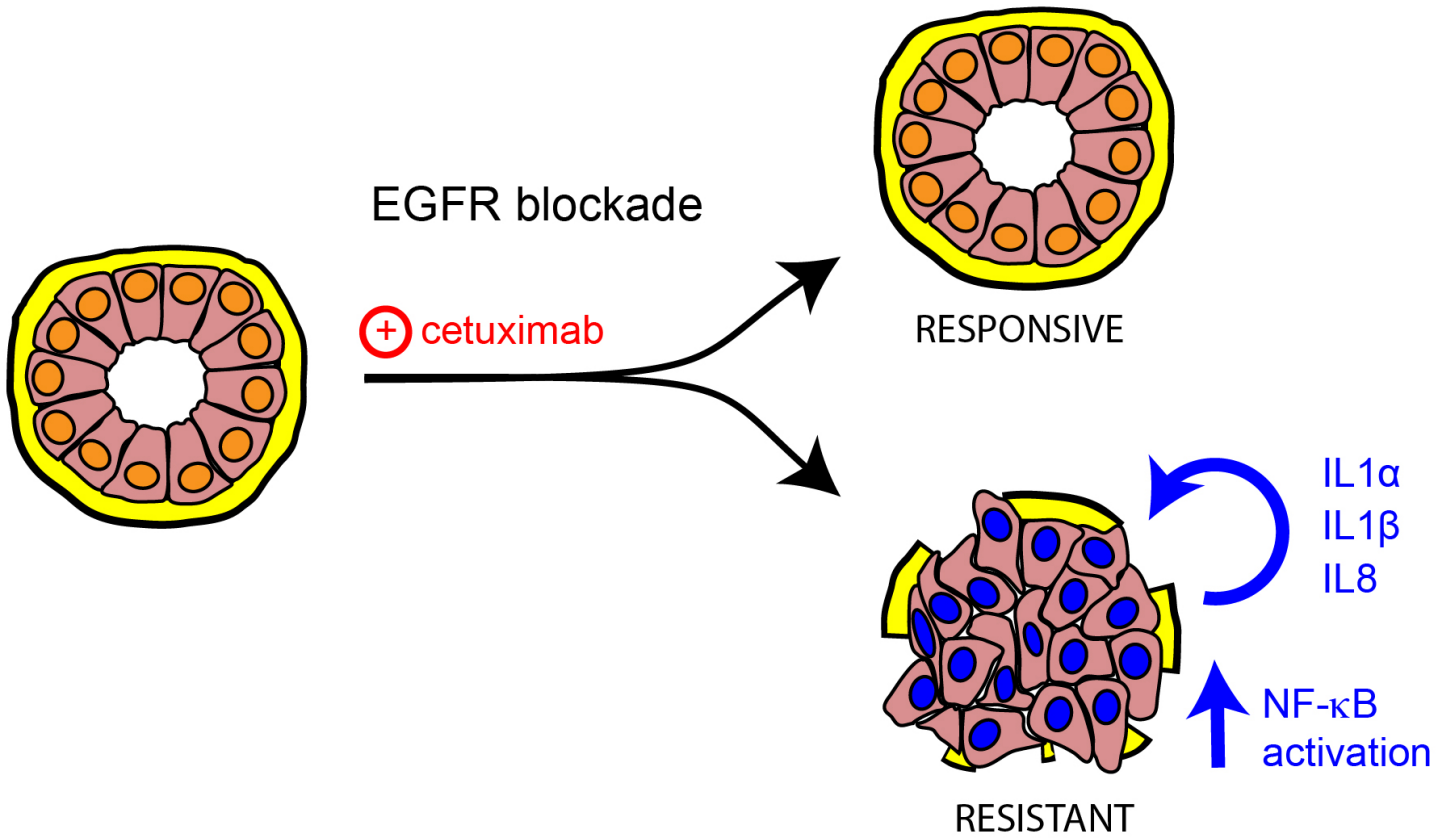
Model depicting the link between the unveiled inflammatory cytokines and resistance to cetuximab Schematic diagram showing a new mechanism of resistance to cetuximab in colorectal cancer, relying on autocrine or paracrine production and secretion of the inflammatory cytokines IL1A, IL1B and IL8. Under EGFR targeting, colorectal tumors might display rewiring of cell signalling with consequent undifferentiated phenotype and activation of the indicated cytokines along with NF-kB signalling.

It is notable that cetuximab appears to be more effective than EGFR TKIs in cancers with ligand-dependent activation of EGFR, whereas TKIs are more effective in cancers with EGFR mutations. Cetuximab might block and compete with the ligands production [33]. Hence, in colon cancer the autocrine/paracrine action of soluble growth factors might have a central role in mediating drug response [34]. Our study indicates a new mechanism of resistance to cetuximab in mCRC that relies on autocrine or paracrine production and secretion of inflammatory cytokines (Figure 7). Moreover resistant cells display cellular plasticity toward an undifferentiated phenotype, lacking E-cadherin and featuring strong up-regulation of vimentin and *SNAIL*.

Recently, it has been suggested that mutated KRAS clones may emerge in patients during cetuximab treatment over time, thus providing a rationale to understand cancer cell resistance to cetuximab [35]. Interestingly, RAS dependent activation of NF-κB might be responsible for the production of several cytokines [36]. Therefore the increase in *IL1A*, *IL1B* and *IL8* in resistant cells might represent emergence of the KRAS-mutation. Nevertheless, the sequencing analysis confirmed a KRAS wild type status of our resistant cells, which might suggest that NF-κB activation and cytokines production occurrence is an epiphenomenon, responsible for activation of alternative signalling routes, thus overcoming drug response. The unveiled promotion of a surrounding microenvironment enriched in pro-inflammatory cytokines capable of impairing the cetuximab response in otherwise sensitive cells, certainly represents an emerging link between EGFR and cytokine signalling in colon cancer. These findings are in line with the detection of the rapid engaging of NF-κB signalling upon EGFR inhibitor treatment, which in lung cancer was proved to promote cell survival and resistance to treatment [37]. Importantly, our data clearly show that patients with an alteration in the panel of pro-inflammatory cytokines are not sensitive to cetuximab treatment, recapitulating the trend of KRAS resistant patients. Notably, the administration of IL1 receptor antagonist (Anakinra), in combination with Bevacizumab for mCRC, is currently under evaluation in clinical trial (NCT02090101). Further, IL1A inhibition, by mean of a true humanized antibody (MABp1), showed promising results in refractory cancers [38]. Therefore, blocking these cytokines in combination with EGFR targeting agents might represent a promising therapeutic strategy for colorectal cancer patients. To sum up, these data point to a new approach to treat colorectal patients that poorly respond to EGFR targeted therapies. Indeed, the inhibition of the inflammatory cytokines might push the cells towards a new steady state, sensitive for the EGFR inhibition.

## MATERIALS AND METHODS

### Cell lines and establishment of resistant cells

Caco-2 cells were maintained in DMEM supplemented with 10% fetal bovine serum and 1% Pen/Strep. Caco-2 were cultured adding cetuximab (Erbitux, Merck KgaA, Germany) and gefitinib (ZD1839 Astra Zeneca) at increasing concentrations, up to 10 μg/ml and 10 μM respectively, for a few months, till the resistant cells were established. Cetuximab-resistant cells (CXR) were then maintained in culture with cetuximab (2 μg/ml) and gefitinib-resistant cells (GBR) with gefitinib (1 μM). Parthenolide was purchased from SIGMA, P0667 and diluted in DMSO.

Cells were routinely tested for mycoplasma contamination. MCF10A were kindly provided by Yosef Yarden Laboratories. MCF10A cells were cultured with DMEM/F12 medium (GE Healthcare) as previously described [39] supplemented with 10 μg/ml Insulin (I6634, Sigma), 0.5 μg/ml Cholera Toxin (C8052, Sigma), 0.5 μg/ml hydrocortisone (H0888, Sigma), 5% heat inactivated horse serum and 10ng/ml EGF (E9644, SIGMA). The cells were serum-starved overnight in plain medium and thereafter treated with EGF (10 ng/ml).

### Proliferation assay

Cells were seeded in 96-well plates, 2.000 cells for each well, in 100μl of 5% FBS medium. Quantification of initial time (time 0) was performed the following day using Alamar Blue (20 μM) in medium 0% FBS, measuring the fluorescence after 5 hours of incubation. Fluorescence was quantified using VICTOR^2^_TM_ 1420 multilabel counter (Perkin Elmer, Massachusetts, USA), at a wavelength of 595 nm. Cells were then treated according to the experiment and, after 72-96h proliferation was measured using Alamar Blue, following the same procedure. Data were analysed subtracting background values, normalizing the endpoint values on the initial time ones, calculating for each treatment the median value and transforming it as percentage of the untreated control. Data points represent the median +/− SD. Each experiment was repeated at least three times.

### Colony forming assay

2.000 cells were seeded in 12-well plates in 1 ml of medium. Treatments were added immediately or the following day, according to the information included in the figure legends. After two weeks, the medium was removed, and the cells were washed with PBS and fixed with a solution of methanol and acetone 1:1 for 20 minutes at -20°C. After a washing with PBS, the cells were stained with Crystal Violet 0.5% for 30 minutes, then were washed with water to remove excess dye. A picture of each well was taken and the covered area was measured using ImageJ software. The mean value from covered area values returned by the software was calculated for each treatments and recorded as a percentage of control. Each experiment was repeated at least three times.

### Spheroid assay

To inhibit cellular adhesion to the plastic surface, 6-well plates were covered with a layer of agar 0.6%. Agar 1.8% was autoclaved and diluted to 0.6% with full medium; 1.5 ml of 0.6% agar was used to cover each well and the agar layer was left to dry before seeding cells. 5.000 or 10.000 cells were seeded for each well in 2 ml of 5% FBS medium, supplemented with EGF (10 ng/ml). Treatments were added immediately or the following day, according to the information included in the figure legends. After 10 days of treatment, pictures of four non-overlapping fields for each well were collected using the microscope at 4X magnification. Spheroids of each picture were counted, and the length of the major and minor axis of each spheroid was measured using ImageJ Software. Axis values below 70 A.I. were excluded as not corresponding to mature spheroids and volume was calculated applying the sphere adapted formula (major axis x minor axis)^2^/2. Each experiment was repeated at least three times.

### Spheroids sections and hematoxylin-eosin staining

Spheroids from 3D culture were collected, centrifuged and medium was removed. After PBS washing, spheroids were fixed in formalin 4% for an hour and washed with water. Spheroids were dehydrated with successive steps of five minutes at increasing concentrations of ethanol (70°, 80°, 95°, 100°) then passed twice in xylene for 30 minutes. After drying, the pellet was embedded in paraffin. Sections of 8-10 μm were cut and put on a slide. After overnight at 62° in a dry stove, sections were rehydrated with successive steps of five minutes at decreasing concentrations of ethanol (100°, 95°, 80°, 70°) and washed with water. They were stained with hematoxylin (Carazzi Emallume) and eosin for 10 and 5 minutes, respectively. Sections were dehydrated as spheroids as above and passed three times in xylene for 5 minutes. Cover slip were applied using DXP mounting media and left to dry. Pictures at 4x and 10x were collected by Microscope Leica DM750.

### Confocal microscopy

Spheroids from 3D culture were collected, centrifuged and medium was discarded. After PBS washing, spheroids were fixed with ice cold acetone:methanol (1:1) for 20 min. The spheres were then placed on Fluorodish Cell Culture Dishes, with optical quality glass bottom and directly stained with 50 μg/ml Phalloidin-TRITC Ab (Sigma, P1951) and 0.1μg/ml 4′,6-Diamidino-2-phenylindole (DAPI, Sigma, D9542). The confocal imaging was performed with a Nikon A1-R confocal laser scanning microscope, equipped with a 20× 0.7 NA objective and with 405 and 561 nm laser lines to excite DAPI and TRITC fluorescence signals. Confocal images (resolution: 1024 × 1024 pixels; gray levels: 4096) were obtained by optical sections of the central region of the spheroids. Volume view with 3D rendering was carried out using the NIS Elements Advanced Research software (Nikon).

### Western blot

Caco-2 cells were lysed with Novagen PhosphoSafe Extraction Reagent (EMD Millipore) plus Protease Inhibitors Cocktail (Sigma-Aldrich) and incubated for 10 minutes on ice. Protein concentration in the supernatants was determined by DC Protein Assay (Bio-Rad) using bovine serum albumin as the standard. Proteins (15 μg of total lysate) were separated on 8% polyacrylamide gel and then transferred to polyvinylidene difluoride membranes (Bio-Rad). After blocking with TBS containing 0.1% tween 20 plus 5% non-fat dry milk (Bio-Rad) for one hour at room temperature, membranes were incubated overnight at 4°C with primary antibodies diluted in blocking buffer. The following primary antibodies were used: anti-AKT rabbit polyclonal antibody (1:1000 dilution; #9272, Cell Signaling), anti-phospho-AKT (Ser473) (D9E) XP rabbit monoclonal antibody (1:1000 dilution; #4060, Cell Signaling), anti-p44/42 MAPK (Erk1/2) (137F5) rabbit monoclonal antibody (1:1000 dilution; #4695, Cell Signaling), anti-phospho-p44/42 MAPK (Erk1/2) (Thr202/Tyr204) rabbit polyclonal antibody (1:1000 dilution; #9101, Cell Signaling) (all from Cell Signaling Technology) and anti-actin rabbit polyclonal antibody (1:800; A2066, Sigma Aldrich). Protein presence was detected through the incubation with anti-rabbit horseradish peroxidase-labeled secondary antibody (Santa Cruz Biotechnology) followed by chemiluminescent reaction (Clarity Western ECL Substrate, Bio-Rad).

### Conditioned medium collection

500.000 Caco-2 parental and CXR were cultured in 3 ml of full medium supplemented with EGF (10 ng/ml) or cetuximab (2 μg/ml), alone or in combination (according to the figure legends) for 72 hours. Four different conditioned mediums were then collected, centrifuged at 500g for 5 min to remove death cells and debris and used for treatments. Each experiment was repeated at least three times.

### Nuclear-cytoplasm fractionation

Cells were harvested in hypotonic buffer (10 mM HEPES pH 7.9, 1.5 MgCl_2_, 10 mM KCl, 0.5 mM DTT, 0.5% NP40 and 1 mM sodium vanadate) containing a mixture of protease inhibitors. Nuclei were centrifuged at 12.000 at 4°C for 15 minutes and re-suspended in lysis buffer, followed by a sonication step.

### Cytokine arrays

Human Cytokine Array (R&D Systems, Minneapolis, MN) and chemiluminescence detection reagents were used, and signals were normalized to the loading control.

MCF10A cells were starved overnight for growth factors and thereafter treated for 4 hours with EGF (10ng/ml), dexamethasone, DEX (100nM), alone or in combination. Whole cell lysates were collected and spotted on cytokine arrays, following manufacturer's instructions. Chemiluminescence detection reagents were used to visualize and quantify cytokine levels.

### RNA Isolation and qPCR

RNA was extracted using TRIZOL® Reagent (Invitrogen, Life Technologies), chloroform for separation of three phases, isopropanol for RNA precipitation and ethanol 75% for washing. RNA was resuspended in DEPC water. Total RNA quantity and quality were determined using a NanoDropTM Spectrophotometer (Thermo Fischer Scientific, Waltham, MA, USA). RNA quality was also evaluated with agarose gel 1% electrophoresis, visualizing bands, stained with ethidium bromide, through a Geldoc transilluminator (Biorad, California, USA). cDNA was synthesized using a retrotranscription mix consisting of buffer 5x (Thermo Fischer Scientific, Waltham, MA, USA), dNTP mix 1 mM, oligo dT 5 μM, random examer 5 μM, RiboLock RNAse inhibitor 1 U/μl, RevertAid RT (Thermo Fischer Scientific, Waltham, MA, USA) 0,5 U/μl and water, and incubating for the reaction in a Thermal Cycler (VWR, Pennsylvania, USA). Real-time qPCR analysis was performed with Maxima^TM^ SYBR Green qPCR Master Mix 2X (Fermentas, Thermo Fischer Scientific) in C1000_TM_ Thermal Cycler (Biorad, California, USA); the primer list is shown. qPCR signals (CT) were normalized to beta2-microglobulin (B2M), DDCT was calculated and each gene value was linearized to the time zero using the formula 2^^−DDCT^. Each qPCR run always included a negative control lacking cDNA template, and a positive control of cDNA derived from the HT-29 cell line, which express the gene of interest. Reaction efficiency (E) was calculated from the slope of the standard curve generated from 10-fold serial dilutions of calibrator cDNA, according to the formula: E = [10 (−1/slope)−1] × 100. At the end, B2M normalized data were median centered using Cluster software and the unsupervised hierarchical clustering was generated with Euclidean distance as similarity metric followed by complete linkage and finally visualized as a heatmap using Java Tree View software.

**Table:** Primer list

Gene Nam	5′->3′	Amplicon Bp
FH_CDH1	CAGTACAACGACCCAACCCA	135
RH_CDH1	CACGCTGACCTCTAAGGTGG	
FH_ LRIG1	AGAAGAGTGAAGAGTACAGTG	80
RH_LRIG1	CTGAGAAGAGAGGTAGCTTG	
FH_LRIG3	TCGAATTGAACCGAAACAAG	122
RH_LRIG3	CCAAAAAGCTCCATCCATAAG	
FH_VIMENTIN	TCTACGAGGAGGAGATGCGG	213
RH_VIMENTIN	GGTCAAGACGTGCCAGAGAC	
FH_IL8	CGGAAGGAACCATCTCACTG	116
RH_IL8	AGCACTCCTTGGCAAAACTG	
FH_HBEGF	GCTGTGGTGCTGTCATCTGT	115
RH_HBEGF	TCATGCCCAACTTCACTTTCT	
FH_EGFR	AGTGCCTGAATACATAAACC	112
RH_EGFR	GTAGTGTGGGTCTCTGC	
FH_ERBB2	CCAGCCTGAATATGTGAAC	161
RH_ERBB2	CCCCAAAGGCAAAAACG	
FH_IL1 A	TCTGCACTTGTGATCATGGTTT	72
RH_IL1 A	CACATTGCTCAGGAAGCTAAAAG	
FH_IL1 B	CTGAAAGCTCTCCACCTCCA	106
RH_IL1 B	CCAAGGCCACAGGTATTTTG	
FH_TGFA	GTTTTTGGTGCAGGAGGACA	56
RH_TGFA	CACCAACGTACCCAGAATGG	
FH_ERRFI1	GGAATGAAAGCTACTGGTTG	199
RH_ERRFI1	GTTTTTAAACTCACTGCGAC	
FH_SNAIL	ACCACTATGCCGCGCTCTT	115
RH_SNAIL	GGTCGTAGGGCTGCTGGAA	
FH_LEF1	CCGAAGAGGAAGGCGATTTAGC	187
RH_LEF1	GGTCCCTTGTTGTAGAGGCC	
FH_SOX2	ACCAGCTCGCAGACCTACAT	154
RH_SOX2	TGGAGTGGGAGGAAGAGGTA	

### Statistical analysis

The statistical analyses were performed by using Prism version 6 (GraphPad Sotfware, Inc). The one way or two-way ANOVA were used to test significance of the assays.
